# Supplementary material for: Modelling the Unidentified Abortion Burden from Four Infectious Pathogenic Microorganisms (Leptospira interrogans, Brucella abortus, Brucella ovis, and Chlamydia abortus) in Ewes Based on Artificial Neural Networks Approach: The Epidemiological Basis for a Control Policy
Source: Animals (Basel). 2023 Sep 18;13(18):2955. doi: 10.3390/ani13182955 (PMC10525082; doi:10.3390/ani13182955)
Supplement: Supplementary file 1 [file animals-13-02955-s001.zip › Figure S1.pdf]

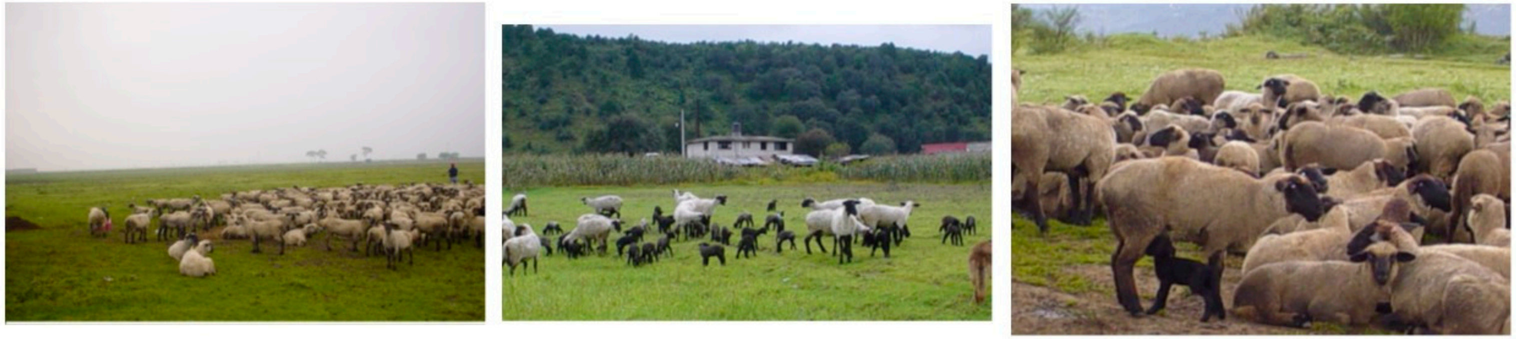

(a) (b) (c)

Figure S1. The view of the flocks of sheep in their habitats where they have shelter, water, and a large grassy pasture. (a) zone 1 (elevations up to 2600 masl.); (b) zone 2 (2601 to 2800 masl.); and (c) zone 3 (>2800 masl.).
