# Supplementary material for: Modelling the Unidentified Abortion Burden from Four Infectious Pathogenic Microorganisms (Leptospira interrogans, Brucella abortus, Brucella ovis, and Chlamydia abortus) in Ewes Based on Artificial Neural Networks Approach: The Epidemiological Basis for a Control Policy
Source: Animals (Basel). 2023 Sep 18;13(18):2955. doi: 10.3390/ani13182955 (PMC10525082; doi:10.3390/ani13182955)
Supplement: Supplementary file 1 [file animals-13-02955-s001.zip › Table S2_Animals.pdf]

Table S2. The geographic distribution of clustered sampling and group size in sheep transhumance systems.

Clusters[&]	Municipalities	Number of animals	Sampled animals	Cumulative percentage	Geographical coordinates		
					Northern latitude	Western longitude	Altitude (masl)
1	Lerma	34	3	.9	19°18′41′′	99°26′23′′	2574
2	Ocoyoacac	22	7	2.9	19°17′05′′	99°26′34′′	2731.2
3	Ocoyoacac	206	8	5.2	19°16′30′′	99°29′11′′	2735
4	Capulhuac	160	8	7.5	19°14′54′′	99°27′15′′	2735.22
5	Lerma	154	9	10.1	19°14′19′′	99°28′53′′	2866
6	Lerma	51	6	11.9	19°14′16′′	99°28′52′′	2572
7	Lerma	182	12	15.4	19°14′16′′	99°28′42′′	2574
8	Capulhuac	76	18	20.6	19°13′18′′	99°27′22′′	2735.05
9	Chapultepec	176	7	22.6	19°12′01′′	99°31′21′′	2574.32
10	Chapultepec	145	6	24.3	19°12′02′′	99°31′21′′	2574.27
11	Santiago Tianguistenco	295	10	27.2	19°12′03′′	99°31′21′′	2574.26
12	Chapultepec	120	16	31.9	19°12′04′′	99°31′21′′	2574.24
13	Santiago Tianguistenco	94	5	33.3	19°12′05′′	99°31′21′′	2574.26
14	Santiago Tianguistenco	111	7	35.4	19°12′06′′	99°31′21′′	2574.27
15	Santiago Tianguistenco	107	13	39.1	19°12′10′′	99°25′48′′	2735.12
16	Capulhuac	120	10	42	19°12′26′′	99°28′18′′	2735.1
17	Santiago Tianguistenco	48	10	44.9	19°12′11′′	99°31′33′′	2575.12
18	Chapultepec	60	10	47.8	19°12′12′′	99°31′37′′	2575.16
19	Chapultepec	150	15	52.2	19°12′13′′	99°31′37′′	2575.11
20	Santiago Tianguistenco	140	10	55.1	19°11′55′′	99°31′21′′	2574.31
21	Santiago Tianguistenco	298	24	62	19°11′56′′	99°31′21′′	2574.34
22	Xalatlaco	130	17	67	19°10′47′′	99°24′25′′	2817
23	Xalatlaco	200	12	70.4	19°10′08′′	99°25′13′′	2827
24	Xalatlaco	76	7	72.5	19°10′03′′	99°25′02′′	2811
25	Xalatlaco	18	10	75.4	19°10′01′′	99°25′08′′	2952
26	Xalatlaco	35	8	77.7	19°09′48′′	99°23′01′′	2995
27	Xalatlaco	121	8	80	19°09′40′′	99°24′15′′	2831
28	Xalatlaco	130	24	87	19°09′20′′	99°24′27′′	2829

29	Tescalyacac	130	10	89.9	19°08′12′′	99°30′56′′	2574.3
30	Tescalyacac	46	7	91.9	19°08′13′′	99°30′56′′	2574.3
31	Tescalyacac	51	9	94.5	19°08′14′′	99°30′58′′	2574.29
32	Tescalyacac	117	10	97.4	19°08′24′′	99°31′06′′	2574.29
33	Tescalyacac	59	3	98.3	19°08′27′′	99°31′25′′	2574.29
34	Tescalyacac	20	4	99.4	19°08′29′′	99°30′48′′	2574.29
35	Tescalyacac	10	2	100	19°08′34′′	99°30′58′′	2574.29

[&]The numbering of the clusters does not correspond to the order of sampling selection.

masl., meters above sea level.
