# Supplementary material for: Modelling the Unidentified Abortion Burden from Four Infectious Pathogenic Microorganisms (Leptospira interrogans, Brucella abortus, Brucella ovis, and Chlamydia abortus) in Ewes Based on Artificial Neural Networks Approach: The Epidemiological Basis for a Control Policy
Source: Animals (Basel). 2023 Sep 18;13(18):2955. doi: 10.3390/ani13182955 (PMC10525082; doi:10.3390/ani13182955)
Supplement: Supplementary file 1 [file animals-13-02955-s001.zip › Table S3_Animals.pdf]

Table S3. Schwarz´s and Akaike´s Bayesian Information Criteria for 28 generalized linear models of infectious abortion in ewes.

Generalized linear models.	BIC[ŧ]	AIC[ŧŧ]	p-value
Grippotyphosa_1 + Portland_1 + Mixed_infections2 + Bed_straw + Mixed_infections3 + Housing1 + Dual_infection6 + Municipality_7 + Municipality_2 + Purchased3 + Hardjo_1 + Pyrogenes_1 + Breed5 + Mixed_infections1 + Dual_infection4 + Drinking + Sheep_pen + Dual_infection5 + Raise + Breed3 + Puchased2 + Municipality_6 + Breed2 + Pomona_1 + Zone3 + Zone2 + Dual_infection1 + Municipality_4 + Babortus + Drainage + Pen_flooring + Zone1 + Birthing_pen + Icterohaemorrhagiae_1 + Breed1 + Canicola_1 + Municipality_5 + Dual_infection3 + Dual_infection2 + Municipality_3 + Bratislava_1 + Purchased1 + Bovis + Isolation + Frecclean + Tarassovi_1 + Municipality_1 + Excreta.	386.5	213.9	$<7x10^{-5}$
Grippotyphosa_1 + Portland_1 + Mixed_infections2 + Bed_straw + Mixed_infections3 + Housing1 + Dual_infection6 + Municipality_7 + Municipality_2 + Purchased3 + Hardjo_1 + Breed5 + Mixed_infections1 + Dual_infection4 + Drinking + Sheep_pen + Dual_infection5 + Raise + Breed3 + Puchased2 + Municipality_6 + Breed2 + Pomona_1 + Zone3 + Zone2 + Dual_infection1 + Municipality_4 + Babortus + Drainage + Pen_flooring + Zone1 + Birthing_pen + Icterohaemorrhagiae_1 + Breed1 + Canicola_1 + Municipality_5 + Dual_infection3 + Dual_infection2 + Municipality_3 + Bratislava_1 + Purchased1 + Bovis + Isolation + Frecclean + Tarassovi_1 + Municipality_1 + Excreta.	380.7	211.9	$<5x10^{-5}$
Grippotyphosa_1 + Portland_1 + Bed_straw + Mixed_infections3 + Housing1 + Dual_infection6 + Municipality_7 + Municipality_2 + Purchased3 + Hardjo_1 + Breed5 + Mixed_infections1 + Dual_infection4 + Drinking + Sheep_pen + Dual_infection5 + Raise + Breed3 + Puchased2 + Municipality_6 + Breed2 + Pomona_1 + Zone3 + Zone2 + Dual_infection1 + Municipality_4 + Babortus + Drainage + Pen_flooring + Zone1 + Birthing_pen + Icterohaemorrhagiae_1 + Breed1 + Canicola_1 + Municipality_5 + Dual_infection3 + Dual_infection2 + Municipality_3 + Bratislava_1 + Purchased1 + Bovis + Isolation + Frecclean + Tarassovi_1 + Municipality_1 + Excreta.	380.7	211.9	$<5x10^{-5}$
Grippotyphosa_1 + Portland_1 + Bed_straw + Mixed_infections3 + Housing1 + Dual_infection6 + Municipality_7 + Municipality_2 + Purchased3 + Hardjo_1 + Breed5 + Mixed_infections1 + Dual_infection4 + Drinking + Sheep_pen + Dual_infection5 + Raise + Breed3 + Puchased2 + Municipality_6 + Breed2 + Pomona_1 + Zone3 + Zone2 + Dual_infection1 + Municipality_4 + Babortus + Drainage + Pen_flooring + Zone1 + Icterohaemorrhagiae_1 + Breed1 + Canicola_1 + Municipality_5 + Dual_infection3 + Dual_infection2 + Municipality_3 + Bratislava_1 + Purchased1 + Bovis + Isolation + Frecclean + Tarassovi_1 + Municipality_1 + Excreta.	374.9	209.9	$<2x10^{-5}$

Grippotyphosa_1 + Portland_1 + Bed_straw + Mixed_infections3 + Housing1 + Dual_infection6 + Municipality_7 + Municipality_2 + Purchased3 + Hardjo_1 + Mixed_infections1 + Dual_infection4 + Drinking + Sheep_pen + Dual_infection5 + Raise + Breed3 + Puchased2 + Municipality_6 + Breed2 + Pomona_1 + Zone3 + Zone2 + Dual_infection1 + Municipality_4 + Babortus + Drainage + Pen_flooring + Zone1 + Icterohaemorrhagiae_1 + Breed1 + Canicola_1 + Municipality_5 + Dual_infection3 + Dual_infection2 + Municipality_3 + Bratislava_1 + Purchased1 + Bovis + Isolation + Frecclean + Tarassovi_1 + Municipality_1 + Excreta.	369	207.9	$<2x10^{-5}$
Grippotyphosa_1 + Portland_1 + Bed_straw + Mixed_infections3 + Housing1 + Dual_infection6 + Municipality_7 + Municipality_2 + Purchased3 + Hardjo_1 + Mixed_infections1 + Dual_infection4 + Drinking + Sheep_pen + Dual_infection5 + Breed3 + Puchased2 + Municipality_6 + Breed2 + Pomona_1 + Zone3 + Zone2 + Dual_infection1 + Municipality_4 + Babortus + Drainage + Pen_flooring + Zone1 + Icterohaemorrhagiae_1 + Breed1 + Canicola_1 + Municipality_5 + Dual_infection3 + Dual_infection2 + Municipality_3 + Bratislava_1 + Purchased1 + Bovis + Isolation + Frecclean + Tarassovi_1 + Municipality_1 + Excreta.	363.2	205.9	$<9x10^{-6}$
Grippotyphosa_1 + Portland_1 + Bed_straw + Mixed_infections3 + Housing1 + Dual_infection6 + Municipality_7 + Municipality_2 + Purchased3 + Hardjo_1 + Mixed_infections1 + Dual_infection4 + Drinking + Sheep_pen + Dual_infection5 + Breed3 + Puchased2 + Municipality_6 + Breed2 + Pomona_1 + Zone3 + Zone2 + Dual_infection1 + Municipality_4 + Babortus + Drainage + Pen_flooring + Zone1 + Icterohaemorrhagiae_1 + Breed1 + Canicola_1 + Municipality_5 + Dual_infection3 + Municipality_3 + Bratislava_1 + Purchased1 + Bovis + Isolation + Frecclean + Tarassovi_1 + Municipality_1 + Excreta.	357.4	203.9	$<6x10^{-6}$
Grippotyphosa_1 + Portland_1 + Bed_straw + Housing1 + Dual_infection6 + Municipality_7 + Municipality_2 + Purchased3 + Hardjo_1 + Mixed_infections1 + Dual_infection4 + Drinking + Sheep_pen + Dual_infection5 + Breed3 + Puchased2 + Municipality_6 + Breed2 + Pomona_1 + Zone3 + Zone2 + Dual_infection1 + Municipality_4 + Babortus + Drainage + Pen_flooring + Zone1 + Icterohaemorrhagiae_1 + Breed1 + Canicola_1 + Municipality_5 + Dual_infection3 + Municipality_3 + Bratislava_1 + Purchased1 + Bovis + Isolation + Frecclean + Tarassovi_1 + Municipality_1 + Excreta.	351.5	201.9	$<3x10^{-6}$
Grippotyphosa_1 + Portland_1 + Bed_straw + Housing1 + Dual_infection6 + Municipality_7 + Municipality_2 + Purchased3 + Hardjo_1 + Mixed_infections1 + Dual_infection4 + Drinking + Sheep_pen + Dual_infection5 + Breed3 + Puchased2 + Municipality_6 + Breed2 + Pomona_1 + Zone3 + Zone2 + Dual_infection1 + Municipality_4 + Babortus + Drainage + Pen_flooring + Zone1 + Breed1 + Canicola_1 + Municipality_5 + Dual_infection3 + Municipality_3 + Bratislava_1 + Purchased1 + Bovis + Isolation + Frecclean + Tarassovi_1 + Municipality_1 + Excreta.	345.7	199.9	$<2x10^{-6}$

Grippotyphosa_1 + Portland_1 + Bed_straw + Housing1 + Dual_infection6 + Municipality_7 + Municipality_2 + Hardjo_1 + Mixed_infections1 + Dual_infection4 + Drinking + Sheep_pen + Dual_infection5 + Breed3 + Puchased2 + Municipality_6 + Breed2 + Pomona_1 + Zone3 + Zone2 + Dual_infection1 + Municipality_4 + Babortus + Drainage + Pen_flooring + Zone1 + Breed1 + Canicola_1 + Municipality_5 + Dual_infection3 + Municipality_3 + Bratislava_1 + Purchased1 + Bovis + Isolation + Frecclean + Tarassovi_1 + Municipality_1 + Excreta.	345.7	199.9	$<1x10^{-6}$
Grippotyphosa_1 + Portland_1 + Bed_straw + Housing1 + Dual_infection6 + Municipality_7 + Municipality_2 + Hardjo_1 + Mixed_infections1 + Dual_infection4 + Drinking + Sheep_pen + Dual_infection5 + Breed3 + Puchased2 + Municipality_6 + Breed2 + Pomona_1 + Zone3 + Zone2 + Dual_infection1 + Municipality_4 + Babortus + Drainage + Pen_flooring + Zone1 + Breed1 + Canicola_1 + Municipality_5 + Dual_infection3 + Municipality_3 + Bratislava_1 + Bovis + Isolation + Frecclean + Tarassovi_1 + Municipality_1 + Excreta.	336.7	194.7	$<8x10^{-7}$
Grippotyphosa_1 + Bed_straw + Housing1 + Dual_infection6 + Municipality_7 + Municipality_2 + Hardjo_1 + Mixed_infections1 + Dual_infection4 + Drinking + Sheep_pen + Dual_infection5 + Breed3 + Puchased2 + Municipality_6 + Breed2 + Pomona_1 + Zone3 + Zone2 + Dual_infection1 + Municipality_4 + Babortus + Drainage + Pen_flooring + Zone1 + Breed1 + Canicola_1 + Municipality_5 + Dual_infection3 + Municipality_3 + Bratislava_1 + Bovis + Isolation + Frecclean + Tarassovi_1 + Municipality_1 + Excreta.	329.7	191.5	$<5x10^{-7}$
Grippotyphosa_1 + Bed_straw + Housing1 + Dual_infection6 + Municipality_7 + Municipality_2 + Hardjo_1 + Mixed_infections1 + Dual_infection4 + Drinking + Sheep_pen + Dual_infection5 + Breed3 + Puchased2 + Municipality_6 + Breed2 + Pomona_1 + Zone3 + Zone2 + Dual_infection1 + Municipality_4 + Babortus + Drainage + Pen_flooring + Zone1 + Breed1 + Canicola_1 + Municipality_5 + Dual_infection3 + Municipality_3 + Bovis + Isolation + Frecclean + Tarassovi_1 + Municipality_1 + Excreta.	318.7	184.3	$<3x10^{-7}$
Grippotyphosa_1 + Bed_straw + Dual_infection6 + Municipality_7 + Municipality_2 + Hardjo_1 + Mixed_infections1 + Dual_infection4 + Drinking + Sheep_pen + Dual_infection5 + Breed3 + Puchased2 + Municipality_6 + Breed2 + Pomona_1 + Zone3 + Zone2 + Dual_infection1 + Municipality_4 + Babortus + Drainage + Pen_flooring + Zone1 + Breed1 + Canicola_1 + Municipality_5 + Dual_infection3 + Municipality_3 + Bovis + Isolation + Frecclean + Tarassovi_1 + Municipality_1 + Excreta.	316.2	189.4	$<2x10^{-7}$
Grippotyphosa_1 + Bed_straw + Dual_infection6 + Municipality_7 + Municipality_2 + Hardjo_1 + Mixed_infections1 + Dual_infection4 + Drinking + Sheep_pen +	310.3	187.4	$<1x10^{-7}$

Dual_infection5 + Breed3 + Puchased2 + Municipality_6 + Breed2 + Pomona_1 + Zone3 + Zone2 + Dual_infection1 + Municipality_4 + Babortus + Drainage + Pen_flooring + Zone1 + Canicola_1 + Municipality_5 + Dual_infection3 + Municipality_3 + Bovis + Isolation + Frecclean + Tarassovi_1 + Municipality_1 + Excreta.			
Grippotyphosa_1 + Bed_straw + Dual_infection6 + Municipality_7 + Municipality_2 + Hardjo_1 + Mixed_infections1 + Dual_infection4 + Drinking + Sheep_pen + Dual_infection5 + Breed3 + Puchased2 + Municipality_6 + Pomona_1 + Zone3 + Zone2 + Dual_infection1 + Municipality_4 + Babortus + Drainage + Pen_flooring + Zone1 + Canicola_1 + Municipality_5 + Dual_infection3 + Municipality_3 + Bovis + Isolation + Frecclean + Tarassovi_1 + Municipality_1 + Excreta.	304.1	185.1	<7x10^{-8}
Grippotyphosa_1 + Bed_straw + Dual_infection6 + Municipality_7 + Municipality_2 + Hardjo_1 + Mixed_infections1 + Dual_infection4 + Drinking + Sheep_pen + Dual_infection5 + Breed3 + Puchased2 + Municipality_6 + Pomona_1 + Zone3 + Zone2 + Dual_infection1 + Municipality_4 + Babortus + Drainage + Pen_flooring + Zone1 + Canicola_1 + Municipality_5 + Dual_infection3 + Municipality_3 + Bovis + Frecclean + Tarassovi_1 + Municipality_1 + Excreta.	297.5	182.3	<4x10^{-8}
Grippotyphosa_1 + Bed_straw + Dual_infection6 + Municipality_7 + Municipality_2 + Hardjo_1 + Dual_infection4 + Drinking + Sheep_pen + Dual_infection5 + Breed3 + Puchased2 + Municipality_6 + Pomona_1 + Zone3 + Zone2 + Dual_infection1 + Municipality_4 + Babortus + Drainage + Pen_flooring + Zone1 + Canicola_1 + Municipality_5 + Dual_infection3 + Municipality_3 + Bovis + Frecclean + Tarassovi_1 + Municipality_1 + Excreta.	297.5	182.3	<3x10^{-8}
Grippotyphosa_1 + Bed_straw + Dual_infection6 + Municipality_7 + Municipality_2 + Hardjo_1 + Dual_infection4 + Drinking + Sheep_pen + Dual_infection5 + Breed3 + Puchased2 + Municipality_6 + Pomona_1 + Zone3 + Zone2 + Dual_infection1 + Municipality_4 + Babortus + Drainage + Pen_flooring + Zone1 + Canicola_1 + Municipality_5 + Dual_infection3 + Municipality_3 + Bovis + Tarassovi_1 + Municipality_1 + Excreta.	291.7	180.3	<3x10^{-8}
Grippotyphosa_1 + Bed_straw + Dual_infection6 + Municipality_7 + Municipality_2 + Hardjo_1 + Dual_infection4 + Drinking + Sheep_pen + Dual_infection5 + Breed3 + Puchased2 + Municipality_6 + Pomona_1 + Zone3 + Zone2 + Municipality_4 + Babortus + Drainage + Pen_flooring + Zone1 + Canicola_1 + Municipality_5 + Dual_infection3 + Municipality_3 + Bovis + Tarassovi_1 + Municipality_1 + Excreta.	286.3	178.7	<2x10^{-8}
Grippotyphosa_1 + Bed_straw + Dual_infection6 + Municipality_7 + Municipality_2 + Hardjo_1 + Dual_infection4 + Drinking + Sheep_pen + Dual_infection5 + Breed3 + Puchased2 + Municipality_6 + Pomona_1 + Zone3 + Zone2 + Municipality_4 +	281	177.3	<10x10^{-9}

Babortus + Pen_flooring + Zone1 + Canicola_1 + Municipality_5 + Dual_infection3 + Municipality_3 + Bovis + Tarassovi_1 + Municipality_1 + Excreta.			
Grippotyphosa_1 + Bed_straw + Dual_infection6 + Municipality_7 + Municipality_2 + Hardjo_1 + Dual_infection4 + Drinking + Sheep_pen + Dual_infection5 + Breed3 + Puchased2 + Municipality_6 + Pomona_1 + Zone3 + Zone2 + Municipality_4 + Babortus + Pen_flooring + Zone1 + Canicola_1 + Municipality_5 + Municipality_3 + Bovis + Tarassovi_1 + Municipality_1 + Excreta.	270.2	170.3	$<10x10^{-9}$
Grippotyphosa_1 + Bed_straw + Dual_infection6 + Municipality_7 + Municipality_2 + Hardjo_1 + Dual_infection4 + Drinking + Sheep_pen + Dual_infection5 + Breed3 + Puchased2 + Municipality_6 + Pomona_1 + Zone3 + Zone2 + Municipality_4 + Pen_flooring + Zone1 + Canicola_1 + Municipality_5 + Municipality_3 + Bovis + Tarassovi_1 + Municipality_1 + Excreta.	249.9	153.9	$<10x10^{-9}$
Grippotyphosa_1 + Bed_straw + Dual_infection6 + Municipality_7 + Municipality_2 + Hardjo_1 + Dual_infection4 + Drinking + Sheep_pen + Breed3 + Puchased2 + Municipality_6 + Pomona_1 + Zone3 + Zone2 + Municipality_4 + Pen_flooring + Zone1 + Canicola_1 + Municipality_5 + Municipality_3 + Bovis + Tarassovi_1 + Municipality_1 + Excreta.	235.4	143.3	$<10x10^{-9}$
Grippotyphosa_1 + Bed_straw + Dual_infection6 + Municipality_7 + Municipality_2 + Hardjo_1 + Dual_infection4 + Drinking + Sheep_pen + Breed3 + Puchased2 + Municipality_6 + Pomona_1 + Zone3 + Zone2 + Municipality_4 + Pen_flooring + Zone1 + Municipality_5 + Municipality_3 + Bovis + Tarassovi_1 + Municipality_1 + Excreta.	219.4	131	$<10x10^{-9}$
Grippotyphosa_1 + Bed_straw + Dual_infection6 + Municipality_7 + Municipality_2 + Hardjo_1 + Dual_infection4 + Drinking + Sheep_pen + Breed3 + Puchased2 + Municipality_6 + Zone3 + Zone2 + Municipality_4 + Pen_flooring + Zone1 + Municipality_5 + Municipality_3 + Bovis + Tarassovi_1 + Municipality_1 + Excreta.	210.7	126.3	$<10x10^{-9}$
Grippotyphosa_1 + Bed_straw + Dual_infection6 + Municipality_7 + Municipality_2 + Hardjo_1 + Dual_infection4 + Drinking + Sheep_pen + Breed3 + Municipality_6 + Zone3 + Zone2 + Municipality_4 + Pen_flooring + Zone1 + Municipality_5 + Municipality_3 + Bovis + Tarassovi_1 + Municipality_1 + Excreta.	207.6	126.9	$<10x10^{-9}$
Grippotyphosa_1 + Bed_straw + Municipality_7 + Municipality_2 + Hardjo_1 + Dual_infection4 + Drinking + Sheep_pen + Breed3 + Municipality_6 + Zone3 + Zone2 + Municipality_4 + Pen_flooring + Zone1 + Municipality_5 + Municipality_3 + Bovis + Tarassovi_1 + Municipality_1 + Excreta.	201.9	125	$<10x10^{-9}$

[t] Schwarz´s Bayesian Information Criterion
[tt] Akaike´s Information Criterion

Putative factors: Mixed_infections3: *Leptospira spp.-Chlamydia abortus*-smooth *Brucella spp.-B. ovis*; Portland_1: Portland-vere strain; Municipality_2: Santiago Tianguistenco municipality; Breed3: Suffolk-Pelibuey crossbreed; Hardjo_1: Hardjo; Puchased2: Imported animals; Municipality_3:

Calpulhuac municipality; Dual_infection2: *smooth Brucella spp.-Brucella ovis*; Drainage: Drainage in the pen; Breed1: Pelibuey; Pomona_1: Pomona; Mixed_infections2: *Leptospira-Chlamydia-Brucella ovis*; Municipality_5: Texcalyacac municipality; Bratislava_1: Bratislava; Drinking: Drink water from trough; Zone3: >2,800 masl; Zone2: 2,600 to 2,800 masl; Dual_infection6: *Chlamydia abortus-Brucella ovis*; Municipality_1: Xalatlaco municipality; Puchased1: Rural market; Municipality_4: Chapultepec municipality; Grippotyphosa_1: Grippotyphosa; Municipality_7: Ocoyoacac municipality; Pyrogenes_1: Pyrogenes; Puchased3: Born in the flock; Isolation: Isolation of an individual sheep by panic; Icterohaemorrhagiae_1: Icterohaemorrhagiae; Pen_flooring: Sheep pen flooring in dirt pen flooring; Canicola_1: Canicola; Tarassovi_1: Tarassovi; Sheep_pen: Materials for the construction of sheep pens (bricks); Dual_infection1: *Leptospira spp-Chlamydia abortus*; Frecclean: What is the cleaning frequency of sheep housing?; Excreta: What handling of excreta do you carry out in the housing pen?; Municipality_8: Lerma municipality; Municipality_6: Metepec municipality; Birthing_pen: Where was born the lamb of this ewe? (Birthing pen or meadow); Bovis: *Brucella ovis*; Dual_infection5: *Chlamydia abortus*-smooth *Brucella spp.*; Dual_infection3: *Leptospira spp*-smooth *Brucella spp.*; Breed2: Hampshire; Breed4: Hampshire-Pelibuey crossbreed; Mixed_infections1: *Leptospira spp-Chlamydia abortus*-smooth *Brucella spp.*; Zone1: <2,600 masl; Breed5: Suffolk; Bed_straw: Bed straw: Ewe gave birth on a bed of straw?; Babortus: *smooth Brucella spp.*; Housing1: How many animals does it take to congregate to avoid panic? (<10 animals; 10 to 15 animals; >15 animals).
