# Supplementary material for: Modelling the Unidentified Abortion Burden from Four Infectious Pathogenic Microorganisms (Leptospira interrogans, Brucella abortus, Brucella ovis, and Chlamydia abortus) in Ewes Based on Artificial Neural Networks Approach: The Epidemiological Basis for a Control Policy
Source: Animals (Basel). 2023 Sep 18;13(18):2955. doi: 10.3390/ani13182955 (PMC10525082; doi:10.3390/ani13182955)
Supplement: Supplementary file 1 [file animals-13-02955-s001.zip › Table S4_Animals.pdf]

Table S4. Questionnaire to collect information about the flocks, animals, and risks factors related to ewes' abortion.

This form lists a few questions about infectious diseases and some risk factors which be associated with abortion. We place a mark only one in the appropriate box.

1. SHEEP FLOCK DATA:
Owner´s name:____
Municipality: □ *Calpulhuac* □ *Ocoyoacac* □ *Texcalyacac* □ *Chapultepec* □ *Santiago-Tianguistenco* □ *Xalatlaco.*
Sheep breed: □ *Peliguey* □ *Hampshire* □ *Suffolk* □ *Suffolk-Peliguey crossbreed* □ *Hampshire-Pelibuey crossbreed.*
What do you raise sheep for? □ *Market* □ *Breeding program*
Did you make additions to the sheep flock in the last 6 months? □ *No* □ *Yes.*
Where was this sheep purchased? □ *Rural market* □ *Imported animals* □ *Born in flock.*
Place of lambing: □ *Pasture lambing* □ *Shed lambing.*
Grazing time: □ *Permanent* □ *Occasional.*
What type of pasture does sheep eat when they are mobilized from one place to another?
□ *Alpine herbage* □ *Stalks of maize* □ *Stalks of oats.*
How many animals do you move? ____________
The water supply for the animals is: □ *Drinking water* □ *Lake* □ *Irrigation canals.*

2. SHEEP YARD DESIGN AND CONSTRUCTION:
Geographic location of the animal groups: Coordinates of longitude____________, and altitude__________ of the position. Time________Satellites______________
Holding area (m^2): ____________ Number of animals in paddock: ____________
Number of animals housed at night: ____________
What are the materials you use to build your sheep pen? □ *Bricks* □ *Metal sheets and untreated wood.*
Sheep pen flooring: □ *Dirt pen flooring only* □ *Dirt and concrete pen flooring*
Drainage in sheep pen: □ *No* □ *Yes.*

3. HEALTH AND MANAGEMENT:
Ewe gave birth on a bed of straw? □ *No* □ *Yes*
Where was born the lamb of this ewe? □ *Birthing pen* □ *Meadow.*
Cleaning of bedding: □ *No* □ *Yes.*
What is the cleaning frequency of sheep housing? □ *Never* □ *Twice a week.*
What handling of excreta do you carry out in the housing pen? □ *The excreta stay spread out on the meadow* □ *Agglomeration of excreta near the housing pen.*
Removal of aborted fetuses and fetal membranes from meadows: □ *No* □ *Yes.*
Do you use wear gloves or masks to prevent the transmission of microorganisms?
□ *No* □ *Yes.*
When a storm comes, what do you do to avoid animals from panicking? □ *Congregate in a place, only.*
□ *Congregate to avoid panic and give forage with vitamin and mineral supplements.*
Do you set traps to capture rodents or other wildlife? □ *No* □ *Yes.*

4. CLINICAL FEATURES
This checklist is designed for use in each one selected animal. Please, mark with "0= No" or "1=Yes" in the appropriate box.

No.	Abortion/Stillbirth, jaundiced/Haemorrhagic fetus	Haemoglobinuria	Jaundice	Haemorrhages	Anaemia	Conjunctivitis	Mastitis (Black udder)	Panic	Depression or sleepy sickness

5.OTHERS DISEASES AND CONDITIONS

Please tell us, how serious you consider each of problems that there are in your animals.

Diseases/syndromes	Serious problem	Moderate problem	Present but no problem	Non-existent
**Infertility**				
**Bearing troubles (Prolapse of vagina)**				
**Lambing difficulties**				
**Mastitis (Black udder)**				
**Diarrhea in lambs**				
**Arthritis in lambs**				
**Lamb deaths during first two weeks of life**				
**Facial eczema**				
**Pneumonia**				
**Salmonellosis (Diarrhea)**				
**Pinkeye**				
**Pregnancy toxaemia**				
